# Beyond performance: the role of illness perception and internal comparison standards in patient-reported physical function assessments

**DOI:** 10.1186/s41687-026-01227-6

**Published:** 2026-10-08

**Authors:** Nina Schnaderbeck, Mia Grote, Sandra Nolte, Audrey Y. Brinker, Alexander Obbarius, Felix Fischer, Matthias Rose, Gregor Liegl

**Affiliations:** 1 Department of Psychology, Medical School Berlin, Berlin, Germany; 2 Department of Psychosomatic Medicine, Center for Patient-Centered Outcome Research, Charité – University Medical Center Berlin, Corporate Member of Freie Universität Berlin and Humboldt-Universität zu Berlin, Berlin, Germany; 3 Department of Psychology, Friedrich-Alexander-Universität Erlangen-Nürnberg, Erlangen, Germany; 4 Faculty of Medicine, Person-Centered Research, Eastern Health Clinical School, Monash University, Melbourne, Australia

**Keywords:** Physical function, Patient-reported outcome, Performance-based outcome, Illness perception, Social comparison, Temporal comparison, Internal comparison standard

## Abstract

**Background:**

Previous research has yielded inconsistent findings regarding the relationship between patient-reported physical function (PF) and physical performance. Patient-reported PF has been discussed to be more strongly associated with psychological variables, which could explain discrepancies with performance assessments. The aim of this study was to investigate differential association patterns of illness perception and internal comparison standards with patient-reported versus performance-based PF.

**Methods:**

A total of *N* = 106 patients from three medical specialties (Cardiology, *n* = 22; Rheumatology, *n* = 21; Psychosomatic medicine, *n* = 63) were included in this cross-sectional study. Patient-reported PF was assessed using a 20-item short form of the Patient-Reported Outcomes Measurement Information System (PROMIS), the PROMIS-PF SF-20a, while performance-based PF was measured using the Physical Performance Test (PPT), comprising 9 physical performance tasks. Illness perception was assessed using the Brief Illness Perception Questionnaire (BIPQ), while social and temporal comparisons were assessed using single-item scales. Multiple linear regression analyses were conducted to examine the relationships of illness perception and internal comparison standards with both patient-reported and performance-based PF.

**Results:**

Physical performance was strongly associated with patient-reported PF (Pearson’s *r* = .74), accounting for more than 50% of the variance in the regression model with patient-reported PF as dependent variable. Illness perception and internal comparison standards also contributed significantly to the prediction of patient-reported PF beyond actual performance. Specifically, more negative illness perceptions, as well as poorer self-assessments compared to peers (social comparisons) and compared to one’s own best functioning (temporal comparisons), were associated with lower patient-reported PF. Illness perception was also significantly associated with performance-based PF, however, this association was very small. Social and temporal comparisons were not associated with performance-based PF. Consistent with this pattern, the additional explained variance by psychological variables was substantially larger for patient-reported PF than for performance-based PF.

**Conclusions:**

While patient-reported functioning was highly associated with actual performance, illness perception as well as social and temporal comparisons appeared to play an additional role in the self-assessment of PF. In contrast, psychological variables showed only negligible effects when performance-based PF was used as the outcome. These findings highlight the importance of considering psychological variables when interpreting patient-reported PF. Future research is needed to explore the role of further psychological factors not considered in this study to further improve our understanding of self-reported PF.

**Supplementary Information:**

The online version contains supplementary material available at https://doi.org/10.1186/s41687-026-01227-6.

## Background

The quality of life of people with chronic illnesses is often significantly impaired [1]. Health-related quality of life (HRQoL) is a frequently used concept encompassing physical and psychosocial aspects of one’s health or disease status more comprehensively [2]. A key aspect of HRQoL is physical function (PF), which refers to a person’s ability to perform physical activities of daily living (ADLs) [3]. PF is an important parameter in various medical fields, as it is associated with poor health and increased mortality in chronic illnesses (e.g. cardiac disease) [4, 5].

Patient-reported outcomes (PROs) and performance outcomes (PerfOs) are the most commonly used assessment types to measure PF [6, 7]. PRO measures, which are typically standardized questionnaires with closed-response formats, assess PF by evaluating an individual´s perceived ability to perform a variety of physical activities and ADLs [8]. In contrast, PerfOs rely on the actual performance of physical tasks [6]. These tests are often conducted by trained examiners according to standardized protocols, making them a more objective method to assess PF. However, PerfOs are also more resource intensive compared to PROs [9].

There is currently no general recommendation regarding which type of measure should be used to assess PF. While several previous studies found that PROs and PerfOs are only moderately correlated [7, 10–12], more recent research has also shown that both assessment types exhibit a high level of agreement at the group level when the item content is broad, reflecting general physical function, and consistent across assessment types [7, 10, 13, 14]. However, at the individual patient level, research has demonstrated that PROs may be more influenced by subjective patient variables (e.g., pain) than actual performance [13, 15, 16]. This raises the possibility that psychological factors help explain why PROs and PerfOs diverge at the individual level in some cases. In line with this, previous research on the relationship between psychological variables and PF has been conducted, with the majority of studies focusing on PROs [20–25]. However, performance-based assessments of PF are not entirely free from subjectivity. Factors such as fear of pain or injury, as well as low motivation, may influence test performance [3, 17, 18]. Studies that examine both PROs and PerfOs in unison are still rare. For example, potentially relevant psychological variables, including illness perception and internal comparison standards, have yet to be explored in terms of their differential impact on subjective versus objective functioning.

According to the HRQoL model by Ferrans et al. (2015) [3], which is a revised version of the prominent model by Wilson and Cleary (1995) [19], individual psychological characteristics play a crucial role in self-assessing HRQoL, including patient-reported PF. In particular, a person’s illness perception and their individual internal comparison standards, such as social or temporal (with the own past best functioning) comparisons, were identified as relevant intrapersonal factors for subjective PF [3, 10, 20].

*Illness perception* refers to an individual’s cognitive and emotional representations of one’s illness, shaped by personal experiences, beliefs, and symptoms [21]. According to the Common-Sense Model (CSM), people develop mental models of their illness, which influence how they cope with it. Key dimensions of illness perception include beliefs about the cause of one’s disease, timeline, consequences, control, and identity (symptom experience) [22]. These perceptions impact health behaviors, such as treatment adherence and self-management, which are directly linked to PF and, consequently, influence its self-evaluation [23, 24]. Empirical findings highlight that greater perceived control over the disease is associated with better patient-reported PF. In contrast, perceptions of negative consequences, high symptom burden, and an unpredictable disease course correlate with poorer patient-reported PF [25–28]. A negative association was also found between a threatening overall illness perception and poorer patient-reported PF [29].

In the present study, *internal comparison standards* are defined as (1) the comparison to peers at the same age (‘social comparisons’) and (2) the comparison to the patient´s best self-perceived PF in the past (‘temporal comparisons’). Social comparisons, especially in situations of uncertainty and illness, are essential for assessing one’s health status, particularly for individuals with chronic diseases [30]. The social comparison theory suggests that patients with chronic conditions frequently compare themselves with others suffering from similar conditions to better understand their symptoms or coping strategies [21]. Research has shown that this tendency engages in upward comparisons (i.e. comparing oneself to those doing better) can lead to more active coping strategies, lower pain levels, fewer depressive symptoms, and better PF [31, 32]. However, downward comparisons can also have positive outcomes, strengthening self-esteem and enhancing health behaviors, depending on how they are interpreted [33]. In addition, temporal comparisons, where individuals compare their current health status to their past health level, also play a significant role in assessing PF [34]. Previous findings suggest that people evaluate their current PF more negatively when reflecting on their past abilities [35]. This internal comparison can lead to a more critical self-perception, especially for individuals who have experienced a decline in health due to chronic illness. In this context, it is important to consider that internal comparison standards are not necessarily stable over time. Research on patients’ perceived health status and quality of life frequently assumes that such standards remain consistent [28]. However, especially in the face of chronic illness and associated lifestyle changes, internal standards may shift accordingly [24]. These changes, known as *response shift* effects, refer to internal recalibrations that influence how individuals evaluate their health status. The onset of illness can activate various coping mechanisms - such as social or temporal comparisons - which may, in turn, lead to a reassessment of functional abilities [36, 37].

Consequently, PROs may reflect not only actual physical limitations but also psychological adaptation processes. On this basis, the mentioned individual psychological variables, i.e., illness perception and internal comparison standards, were assumed to represent significant determinants of self-reported PF, whereas they are less relevant for actual physical performance assessed under standardized conditions. Accordingly, the present study aimed to examine whether illness perception and internal comparison standards are differentially associated with patient-reported versus performance-based PF when statistically controlling for the respective other assessment type.

## Methods

### Measures

#### Patient-reported outcomes measurement information system physical function

The Patient-Reported-Outcome Measurement Information System (PROMIS) is a psychometrically validated and clinically meaningful measurement system for assessing patient-reported outcomes in various domains such as pain, depression and PF [38, 39]. PROMIS consists of item banks that measure symptoms and aspects of HRQoL. One of the item banks of PROMIS was developed as a generic item bank for assessing PF. The PROMIS PF item bank covers various sub-dimensions of PF and a wide range of physical activities [8]. The different sub-dimensions upper extremity function (dexterity), lower extremity function (walking or mobility), neck and back function, and, activities of daily living such as doing chores. The PROMIS PF item bank has been developed based on item-response theory, which makes it possible to determine a person’s general level of PF using a standardized T-score metric with a general population mean of 50 (*SD* = 10) [3]. Higher T-scores indicate better PF. The present study used the generic 20-item Short Form PROMIS PF SF-20a. PROMIS measures have been translated into various languages and culturally adapted following established guidelines [29]. In this study, the German translation of PROMIS PF SF-20 was used [40]. The psychometric properties of the German versions of PROMIS measures were rated as suitable for both the entire item bank and the short version [40, 41].

#### Physical performance test

The Physical Performance Test (PPT) is a comprehensive PerfO test battery covering various aspects of PF by observing participants’ abilities in simulated activities of daily living. The test comprises nine tasks, each with differing levels of difficulty [42]. Scores of each task range from 0 to 4, mainly depending on how quickly participants complete a task in seconds. A total score can be calculated upon completing all tasks, with higher scores indicating better PF. Like the PROMIS item bank, the PPT covers multiple domains of PF. The nine tasks of the PPT are (1) writing a sentence, (2) simulated eating, (3) lifting a book and putting it on a shelf, (4) putting on and removing a jacket, (5) picking up a coin from the floor, (6) turning 360 degrees, (7) 50-foot-walk test, (8) climbing one flight of stairs, and (9) climbing stairs. The psychometric properties of the scale were rated to be sufficient [42, 43].

#### Illness perception

The Brief Illness Perception Questionnaire (BIPQ) was used to assess illness perception [21]. The BIPQ was developed as a shortened version of the original questionnaire, the Illness Perception Questionnaire-Revised (IPQ-R), which consists of over 80 items [44]. The BIPQ includes eight new items that best summarize the subscales of the original questionnaire. Five of the items assess cognitive illness perceptions: consequences (item 1), timeline (item 2), personal control (item 3), treatment control (item 4), and identity (item 5). Two items assess the emotional representation of the illness: concern (item 6) and emotions (item 7). One item evaluates the understanding of the illness (item 8). The assessment of causal representation is conducted through an open-ended question, asking patients to list the three most important causal factors for their illness (item 9). Item 9 was not assessed in this study - although it may be helpful in clinical practice, it is not relevant to calculate the overall illness perception score. Each item is rated using a 0–10 response scale. Three items (3, 4 and 7) had to be reversed due to their positive wording so that higher total scores (0–80) indicate a more threatening perception of the illness [7]. Kuiper and colleagues (2022) [45] provide the following cutoff values for the total illness perception score: < 42: low perceived threat; 42–49: moderate perceived threat; ≥ 50: high perceived threat. The BIPQ has been translated into multiple languages, including German. For the present study, the German questionnaire version was used [46]. A meta-analysis examined the psychometric properties of the BIPQ. The analysis included 188 studies on various illnesses across 26 languages and 36 countries, including Germany. The review demonstrated that the BIPQ is a widely used measurement tool with established validity and reliability [47].

#### Internal comparison standards

Two questions were newly developed to assess internal comparison standards [13]. These questions were created based on clinical experience and discussions with patients. The following questions used in the study are only translated conceptually here, while they were originally developed in German.

- *How would you rate your current physical function (i.e.*,* your physical ability to perform daily activities) compared to other people your age?*
- *How would you rate your current physical function compared to when you were at your best physical function?*

Both questions use a single-item scale, with responses rated on a numeric rating scale ranging from 0 to 10, where a score of 10 indicates a significant decline in physical function compared to the respective standard (peers/one´s best physical function), and a score of 0 indicates a markedly better perceived physical function.

### Data collection and sample

This cross-sectional study partly utilized data previously collected by a project funded by the German Research Foundation (DFG) [13]. The original study (*N* = 78) examined the relationship between the PPT and PRO measures of general PF, including PROMIS PF. To address the research question of the present study and achieve the required sample size, additional data were collected. The questionnaires and performance tests described were administered to inpatients at the Charité University Hospital in Berlin, Germany. Data were collected at the Department of Cardiology and Angiology, Rheumatology and Clinical Immunology, and the Department for Psychosomatic Medicine. Despite differences in medical focus, these patient groups share important clinical features: most are affected by chronic health conditions characterized by persistent physical symptoms, functional impairments, and varying levels of psychological distress. All patients aged 18 years or older who were cognitively, emotionally, and physically capable of participating were invited to take part and received written information about the study. Based on the findings of previous studies, a medium effect size was assumed [25, 29]. The calculation was performed using G*Power [48]. A final sample size of *N* = 103 was calculated to be sufficient to detect a medium effect size of f^2^= 0.15 in a linear multiple regression model (α = 0.05, power = 0.80). The additional data were subsequently collected at the Department of Psychosomatic Medicine. Following informed consent each participant completed the self-assessment and the performance-based measurement of PF subsequently at the same day.

### Statistical analysis

In a first step, the association between patient-reported PF and potential predicting variables using Pearson correlation was conducted. In a second step, two multiple linear regression models were estimated. The first regression model was estimated to examine the association of patient-reported PF (dependent variable) with illness perception, internal comparison standards (social and temporal comparison), and performance-based PF (independent variables). In the second model, performance-based PF was entered as the dependent variable, with illness perception, internal comparison standards (social and temporal comparison), and patient-reported PF as independent variables. Since a large proportion of the patients exhibited psychosomatic conditions, a third step was conducted, including depressive symptoms – as assessed with the PHQ-2 [46] – as an additional independent variable to the regression models.

A basic model was calculated controlling for potential confounders (i.e., age, sex, and medical specialty: cardiology, rheumatology, psychosomatic medicine). These variables were included as they may also be associated with patient-reported PF and performance-based PF and could otherwise bias the results. Subsequently, the main predictors of interest (performance-based PF/patient-reported PF, illness perception, and social and temporal comparisons) were added sequentially. Illness perception was included as an overall score, representing a continuum from non-threatening to threatening illness perceptions [21]. An alpha level of 0.05 was used for significance testing, and standardized regression coefficients were calculated. The effect size is reported as f^2^, with values of 0.02, 0.15, and 0.35 considered as indicative of small, medium and large effects, respectively [49].

Before examining the assumed correlations, assumptions of multiple linear regression analysis were checked: linearity via bivariate scatterplots, extreme outliers via Cook’s distance (CDi; cutoff > 1), and multicollinearity via intercorrelation tables (cutoff > 0.70). Residual normality and homoscedasticity were checked graphically. Statistical analyses were performed using IBM SPSS Statistics, Version 28.0.1.1.

## Results

### Sample characteristics and descriptive statistics

The total dataset included *N* = 117 participants. Eleven cases (9.4%) with missing values in illness perception and internal comparison standards were excluded listwise. Data imputation was not performed because the missing values were deemed not missing at random, and imputing them could have introduced bias or uncertainty into the analysis of these key variables. The final sample consisted of *N* =106 patients, with 20% (21) from rheumatology, 21% (22) from cardiology, and 59% (63) from psychosomatic medicine. The mean age was 48 years, ranging from 18 to 80 years, and 70 (66%) participants were female. Almost all participants (94%) reported having at least one chronic illness. A more detailed overview of patient characteristics is presented in Table 1.

**Table 1:** Sample characteristics (*N* = 106)

Variables		Descriptive statistics
Mean age (SD)		47.9 (16.9)
Female, n (%)		70 (66)
Male, n (%)		36 (34)
German as native language, n (%)		100 (94)
Professional qualification n (%)		87 (82)
**Medical specialty °**,** n (%)**		
Cardiology		22 (21)
Rheumatology		21 (20)
Psychosomatic medicine		63 (59)
Chronic condition*,** n (%)**		
Any		100 (94)
Rheumatoid arthritis		19 (18)
Back pain		44 (42)
Cardiovascular conditions/problems with the heart		31 (29)
Asthma/severe respiratory illness		14 (13)
Cancer		5 (5)
Depression or anxiety disorder		45 (43)
Diabetes		11 (10)
Stroke		1 (0.9)
Other		53 (50)
Mean Illness duration (years, SD)		2.9 (0.80)
Depressive mood ^+^, mean (SD)		2.6 (1.7)
Positive depressive screen, n (%)*		45,3%

* Based on a patient questionnaire administered to the participants. Patients were asked whether they had one or more of the chronic conditions mentioned in the table. SD indicates standard deviation

* PHQ-2 ≥ 3 indicates a positive screen for probable major depression [54]

+ As measured by the Patient Health Questionnaire-2 with a minimum 0 and a maximum of 6; higher scores indicate more severe depressive symptoms

The characteristics of the variables of patient-reported PF (PROMIS-PF20a), performance-based PF (PPT), illness perception (BIPQ) as well as social and temporal comparison are presented in Table 2. Measure of patient-reported PF resulted in a mean T-score of approximately 40, which is one standard deviation lower than the average self-reported functioning level of the general population. The sum score of the PPT, ranging between 0 and 36, resulted in a mean value of approximately 26. Regarding illness perception, patients had an average score of 47. According to Kuiper et al. (2022) [45], the patients in this study show a moderate level of illness-related threat perception on average. The sample of this study showed a similar level of illness perception, that was observed in previous studies [29, 45]. The results of social and temporal comparisons, indicate that, on average, patients perceived to have slightly lower PF levels compared to peers and their own best physical condition.

**Table 2:** Descriptive statistics of relevant variables

Variables	Metric	Mean (SD)	Range
PROMIS PF SF-20a	T-score	40.7 (8.4)	17.9–62.5
PPT	Sum score (range 0–36)	26.6 (7.7)	5–36
Illness perception	Sum score (range 0–80)	47.6 (11.4)	20–75
Social comparisons	0–10 scale	5.8 (2.4)	0–10
Temporal comparisons	0–10 scale	6.8 (2.9)	0–10

PROMIS indicates Patient-Reported Outcomes Measurement Information; PPT,

Physical Performance Test; SD, standard deviation

### Predictors of patient-reported and performance-based PF

The assumptions for calculating Pearson´s correlation coefficient and multiple linear regression model were fulfilled.

**Fig. 1:**
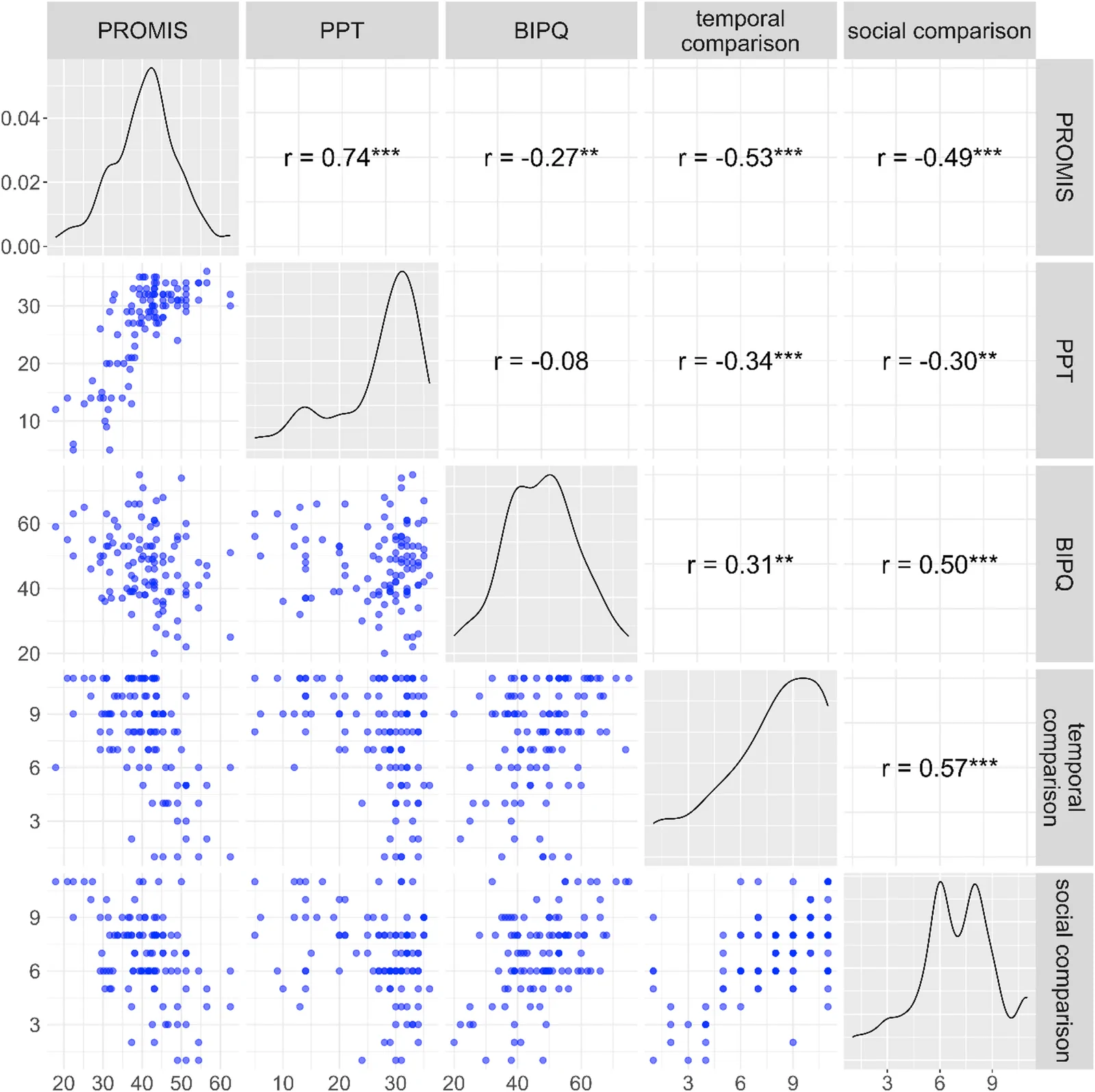
Pearson correlation and scatter plots for all assessed variables and density plots visualizing the distributions of the variables. *Note* * *p* < .05. ** *p* < .01. *** *p* < .001. Corr indicates correlation; PPT, Physical Performance Test; PROMIS, Patient-Reported Outcomes Measurement Information System SF-20a, BIPQ, Brief Illness Perception Questionnaire

The correlation analyses revealed a high correlation between patient-reported and performance-based PF (*r* = .74, *p* < .001). Illness perception (*r* = − .27, *p* < .01) as well as social (*r* = − .49, *p* < .001) and temporal comparisons (*r* = − .53, *p* < .001) were more strongly associated with patient-reported PF than with performance-based PF (Fig. 1).

The results of multiple linear regression analysis for patient-reported PF (*N* = 106) are presented in Table 3. The basic model, controlling for age, gender, and medical specialty as potential confounders (adjusted R^2^ = 12%), indicated that only age (β = − 0.30, *p* = .003) contributed a statistically significant amount of variance to patient-reported PF. When the four predictors of interest (performance-based PF, illness perception, social and temporal comparisons) were included in the model, significantly more variance in patient-reported PF could be explained. As a first step, performance-based PF was added to the model (adjusted R^2^ = 56%), and was found to be a statistically significant predictor of patient-reported PF, with a standardized beta value of β = 0.70 (*p* < .001). Beyond statistical significance, the result also indicates practical relevance, with a large effect size of f² = 1.00. Illness perception was added to the model in the second step (adjusted R^2^ = 63%), with results indicating it to be a highly significant predictor of patient-reported PF (β = − 0.32, *p* < .001), with f² = 0.21, indicating a moderate effect. Finally, the internal comparison standards were added to the model (adjusted R^2^ = 70%), where social comparisons (β = − 0.17, *p* = .025) and temporal comparisons (β = − 0.21, *p* = .003) were also statistically significant. However, the effect sizes were small to medium, with f² = 0.05 for social comparisons and f² = 0.10 for temporal comparisons.

**Table 3:** Results of linear regression model for patient-reported PF (*N* = 106)

Dependent Variable	Predictors (independent variables)	*R*^2^/adjusted *R* ^2^	f^2^		*P* (model)	Standardized beta	*P* (predictor)
PROMIS PF SF-20a	**Basic model**: age, sex, medical specialty	0.153/0.119			0.002		
	**Extended model 1**:	0.576/0.555			< 0.001		
	PPT		1.00			0.700	< 0.001
	**Extended model 2**:	0.650/0.629			< 0.001		
	PPT		1.08			0.666	< 0.001
	Illness perception		0.21			− 0.318	< 0.001
	**Final model**:	0.726/0.704			< 0.001		
	PPT		0.79			0.545	< 0.001
	Illness perception		0.06			− 0.166	0.019
	Social Comparisons			0.05		− 0.171	0.025
	Temporal Comparisons		0.10			− 0.210	0.003

PPT indicates Physical Performance Test; PROMIS, Patient-Reported Outcomes Measurement Information System SF-20a, BIPQ, Brief Illness Perception Questionnaire

The results of the regression model for performance-based PF (*N* = 106) are presented in Table 4. The baseline model (adjusted R² = 11%) showed that both, age (β = − 0.35, *p* < .001) and gender (β = − 0.21, *p* = .03) significantly contributed to the variance in performance-based PF. Subsequently, the predictors of interest were added to the model (patient-reported PF, illness perception, social and temporal comparisons), explaining additional variance in performance-based PF. In the first step, patient-reported PF was added to the model (adjusted R^2^ = 56%) and was found a statistically high significant predictor of performance-based PF (β = 0.73, *p* < .001), with a large effect size of f² = 1.06. When illness perception was added to the model (adjusted R^2^ = 58%), it was also significantly associated with performance-based PF (β = 0.18, *p* = .03), albeit with a small effect (f² = 0.05), compared to the moderate effect observed when predicting patient-reported PF (f² = 0.21). Social (β = 0.04, *p* = .43) and temporal (β = 0.06, *p* = .69) comparisons were not significantly associated with physical performance.

**Table 4:** Results of linear regression model for performance-based PF (*N* = 106)

Dependent Variable	Predictors (independent variables)	*R*^2^/adjusted *R* ^2^	f^2^		*P* (model)	Standardized beta	*P* (predictor)
PPT	**Basic model**: age, sex, medical specialty	0.142/0.108			0.004		
	**Extended model 1**:	0.584/0.563			< 0.001		
	PROMIS PF		1.06			0.729	< 0.001
	**Extended model 2**:	0.604/0.580			< 0.001		
	PROMIS PF		1.13			0.794	< 0.001
	Illness perception		0.05			0.180	0.029
	**Final model**:	0.608/0.576			< 0.001		
	PROMIS PF		0.90			0.844	< 0.001
	Illness perception		0.04			0.158	0.068
	Social Comparisons			0.00		0.040	0.666
	Temporal Comparisons		0.01			0.059	0.495

PPT indicates Physical Performance Test; PROMIS, Patient-Reported Outcomes Measurement Information System SF-20a, BIPQ, Brief Illness Perception Questionnaire

When depressive symptoms were added to the regression models, the explained variance increased only marginally (ΔR² = 0.02 and ΔR² = 0.004, respectively) and neither illness perception nor depressive symptoms remained statistically significant predictors (see Appendix Tables 5 and 6). With regard to self-reported PF, correlation analysis indicated that both variables showed modest bivariate associations with PROMIS PF (*r* = .27) but were strongly intercorrelated (*r* = .55).

## Discussion

This study aimed to examine the role of psychological variables, particularly illness perception as well as social and temporal comparisons, in patient-reported and performance-based PF assessments.

Performance-based PF was found as a highly significant determinant of patient-reported PF. Additionally, illness perception, as well as social and temporal comparisons, were significantly associated with patient-reported PF. In contrast, the association of these psychological variables with observed physical performance was negligible. These results suggest that patients do not only rely on their actual physical performance when assessing their PF but that individual psychological factors also seem relevant.

Given the substantial proportion of the sample presented with psychosomatic conditions, an additional analysis examined whether depressive symptoms influenced the observed associations. When depressive symptoms were added to the regression models, the increase in explained variance was minimal, and neither illness perception nor depressive symptoms were statistically significant predictors. Although both variables showed modest bivariate associations with self-reported PF, they were strongly intercorrelated. This pattern is consistent with overlap between the constructs rather than with the absence of relevant associations for either construct. Given that the primary aim of the study was to investigate illness perception, and because illness perception is conceptually central to the theoretical model guiding this work, illness perception retained as the focal psychological variable in the main analyses. Future studies with larger samples and methods that separate shared from unique variance are needed to disentangle their relative contributions.

The results are in line with previous findings, that PROs are influenced by cognitive and emotional representations of illness and by the reference standards individuals use when judging their abilities [25–28, 31, 35]. Consequently, patients with comparable levels of objective performance may differ in how they perceive and report their functioning, depending on their illness perceptions and internal comparison standards. This emphasizes that variation in PROs does not only reflect differences in functional capacity but may partly stem from differences in cognitive appraisal. Accounting for these influences is therefore essential for accurate interpretation of PROs and for improving comparability across studies using different PF assessment approaches. This interpretation becomes particularly evident when the findings are considered in relation to the regression analysis using performance-based PF as the dependent variable, where psychological variables, in particular illness perception, showed only negligible effects. In line with this, the increase in explained variance was minimal for performance-based PF (ΔR² = 2%), compared to a substantially larger increase for patient-reported PF (ΔR² = 14%). This pattern reinforces the view that subjective evaluations of PF are more strongly influenced by individual psychological processes than objectively measured performance.

The finding that a more threatening overall illness perception is associated with lower self-reported PF aligns with previous research findings [29]. Patient-reported PF seems to be shaped not only by actual physical capacity, but also by the individual assessment and perception of those affected. This perception can be strongly influenced by personal beliefs, emotions, and cognitive processes, which are closely linked to illness perception [50] due to their subjective nature.

Chronically ill patients face the challenge of accepting new life circumstances and coping with the constant threats to their health. Comparisons with others can impact psychological and physical well-being [30]. The results of this study support this assumption. When internal comparison standards were added to the regression model, social and temporal comparisons emerged as statistically significant negative predictors of patient-reported PF. Patients who perceived their PF as worse compared to their peers or compared to their own best PF reported lower PF – independent from actual physical performance. Previous research in this context suggests frequent social comparisons may be associated with a more negative self-assessment of PF [51]. Moreover, the impact of social comparison on evaluating one’s PF seems to depend on how the comparison is perceived [52]. Both upward and downward comparisons can lead to positive or negative self-evaluation. Previous studies have shown that negative social comparisons are more likely to result in passive coping strategies, such as avoidance behavior, which can negatively affect PF [31]. Since the patients in this study are living with at least one chronic illness and have been experiencing functional limitations due to this condition for an extended period of time, it is not surprising that they perceive their current PF as worse compared to their best past PF. This perceived decline in PF may lead to a change in self-perception and, consequently, to a negative self-evaluation [35].

This study has several limitations. Its cross-sectional design precludes the observation of temporal changes, which is particularly relevant in chronic illnesses where PF and illness perception can fluctuate considerably due to daily variations (e.g., pain) [53, 45, 46]. The single-time measurement also prevents causal inferences, allowing only for associations. Moreover, due to the subjective nature of patient-reported PF, it is possible that it may be influenced by other relevant subjective variables which were not considered in this study, such as social desirability, mood, or pain perception [31]. Future research should therefore systematically investigate the impact of these variables to better understand their potential confounding effects. A potential nonresponse bias must be considered, as individuals in poorer health may have been less likely to participate, whereas those with higher motivation might be overrepresented. This could affect the representativeness of the findings. Additionally, listwise exclusion of eleven cases due to missing data may have introduced bias, as missingness might not be completely random (e.g., missing responses might be related to the variable itself). Participants were asked to compare their PF to peers and one´s own best PF, which raises concerns about whether such comparisons are typically made and whether the observed correlation resulted from reactivity to the survey question. Moreover, the measures used for internal comparison standards were not validated, and the direction of social comparison (upward or downward) was not assessed, limiting interpretability [44]. Finally, generalizability is constrained by the sample’s composition, particularly the high proportion of female participants (67%) and the uneven distribution across medical departments. However, controlling’s for variables, such as gender and specialty, should have minimized the risk of substantial bias.

## Conclusion

PF is a key component of HRQOL, making it essential for researchers, clinicians and therapists to understand the factors that may contribute to patient’s self-assessment. The study’s findings highlight that beyond actual physical capacity, illness perception and patients’ internal comparison standards also play a role in patient-reported PF, whereas their influence on performance-based PF was comparatively small. Researchers and clinicians should consider that various psychological variables can influence measurement outcomes when selecting an appropriate assessment method for PF or also when interpreting results. When comparing different measurement methods that assess PF, researchers should consider that illness perception may influence patient-reported PF and may therefore need to be taken into account when interpreting or analyzing these measures. As internal comparison standards showed only small effects further research is needed to better understand the relevance of social and temporal comparisons concerning PF. Nonetheless, the results suggest that temporal comparisons, in particular, are relevant to overall PF, and special attention should be paid to this aspect. Patient-reported and performance-based assessments measure different aspects of PF. Therefore, combining both methods in clinical practice may provide a more comprehensive picture of a patient’s physical abilities. This study supports the revised HRQOL model proposed by Ferrans et al. [3]. Their assumption regarding the importance of individual characteristics in HRQOL was reinforced by identifying additional intrapersonal variables associated with its assessment, such as illness perception as well as social and temporal comparisons.

## Supplementary Information

Below is the link to the electronic supplementary material.

Supplementary Material 1

## Data Availability

The datasets used and/or analysed during the current study are available from the corresponding author on reasonable request.

## Declarations

### Use of artificial intelligence

The authors used AI-assisted tools (ChatGPT, OpenAI) exclusively for language editing and style enhancement. The scientific content, data analysis, and interpretation were entirely developed by the authors.
