# Supplementary material for: Beyond performance: the role of illness perception and internal comparison standards in patient-reported physical function assessments
Source: J Patient Rep Outcomes. 2026 Oct 8;10:186. doi: 10.1186/s41687-026-01227-6 (PMC13653012; doi:10.1186/s41687-026-01227-6)
Supplement: Supplementary file 1 [file 41687_2026_1227_MOESM1_ESM.docx]

**Appendix**

**Table 5** Results of linear regression model for patient-reported PF including depressive mood (*N* = 106).

| Dependent Variable | Predictors  (independent variables) | R^2^/adjusted  R^2^ | f^2^ | | P  (model) | Standardized  beta | P (predictor) |
| --- | --- | --- | --- | --- | --- | --- | --- |
| PROMIS  PF SF-20a | **Basic model:**  age, sex, medical specialty | 0.169/0.136 |  | | < .001 |  |  |
|  | **Extended model:** | 0.747/0.724 |  | | < .001 |  |  |
|  | PPT |  | 0.92 | |  | 0.558 | < .001 |
|  | Illness perception |  | 0.02 | |  | - 0.107 | .163 |
|  | Social Comparison |  | 0.09 | |  | - 0.210 | .005 |
|  | Temporal Comparison |  | 0.05 | |  | - 0.152 | .029 |
|  | Depressive mood |  | | 0.03 |  | - 0.119 | .077 |

PPT indicates Physical Performance Test; PROMIS, Patient-Reported Outcomes Measurement Information System SF-20a.

**Table 6** Results of linear regression model for performance-based PF including depressive mood (*N* = 106).

| Dependent Variable | Predictors  (independent variables) | R^2^/adjusted  R^2^ | f^2^ | | P  (model) | Standardized  beta | P (predictor) |
| --- | --- | --- | --- | --- | --- | --- | --- |
| PPT | **Basic model:**  age, sex, medical specialty | 0.142/0.108 |  | | .004 |  |  |
|  | **Extended model:** | 0.612/0.575 |  | | < .001 |  |  |
|  | PROMIS |  | 0.92 | |  | 0.858 | < .001 |
|  | Illness perception |  | 0.02 | |  | 0.120 | .209 |
|  | Social Comparison |  | 0.00 | |  | 0.056 | .552 |
|  | Temporal Comparison |  | 0.00 | |  | 0.048 | .584 |
|  | Depressive mood |  | | 0.01 |  | 0.079 | .345 |

PPT indicates Physical Performance Test; PROMIS, Patient-Reported Outcomes Measurement Information System SF-20a.
